# Supplementary material for: Complexome profiling of the Chlamydomonas psb28 mutant reveals TEF5 as an early PSII assembly factor
Source: Plant Cell. 2025 Mar 17;37(6):koaf055. doi: 10.1093/plcell/koaf055 (PMC12164590; doi:10.1093/plcell/koaf055)
Supplement: koaf055_Supplementary_Data [file koaf055_supplementary_data.zip › psb28_interactive.zip!/plots/intensity/qualityPlots/peptides/RPL13_Cre14.g630100.t1.2.html]

### Cre14.g630100.t1.2

Trivial name: RPL13  
SeqCoverage [median]: 12.80  
Mass: 24.48 kDa  
  
 

| Sequence | MissedCleavage | Mass [Da] | wtIntensity | muIntensity | Score |
| --- | --- | --- | --- | --- | --- |
| GAVVLPLVK | 0 | 894.59 | 0.00 | 951.62 | 60.29 |
| SLESLQENAQR | 0 | 1273.63 | 0.00 | 546.56 | 109.71 |
| SNLVVFPR | 0 | 930.53 | 1274.72 | 0.00 | 89.75 |
|  |  |  | 1274.7 | 1498.2 |  |
